# Copper Nanocluster‐Decorated Magnesium Silicate‐Based Microneedle Enhances Antimicrobial Effects and Tissue Remodeling for Diabetic Wounds

**DOI:** 10.1002/smsc.202500442

**Published:** 2025-11-19

**Authors:** Shuo Tan, Hua Zeng, Wenshuya Li, Haibo Liu, Xuefeng Gu, Xiong Luo, Xinyu Zhao

**Affiliations:** ^1^ Center for Orthopaedic Science and Translational Medicine Department of Orthopaedics Shanghai Tenth People's Hospital, School of Medicine Tongji University Shanghai 200072 P. R. China; ^2^ Department of Plastic Surgery The Second Hospital of Hebei Medical University Shijiazhuang Hebei 050000 P. R. China; ^3^ Shanghai Key Laboratory of Molecular Imaging School of Pharmacy Shanghai University of Medicine & Health Sciences Shanghai 201318 P. R. China; ^4^ Department of Joint and Sports Medcine Shanghai Fourth People's Hospital, School of Medicine Tongji University Shanghai 200434 P. R. China

**Keywords:** antibacterial microneedles, copper nanoclusters, diabetic wound healing, magnesium silicate nanoparticles, microenvironment remodeling

## Abstract

Diabetic skin lesions, as one of the most common complications of diabetes, present chronic nonhealing wounds that face dual challenges of antibiotic‐resistant bacteria threat and insufficient microenvironment regulation due to hyperglycemic conditions, bacterial infections, and multiple pathological factors (e.g., hypoxia and reactive oxygen species (ROS) accumulation and growth factor deficiency). This study develops a microneedle (MN) system integrated with copper nanocluster‐decorated magnesium silicate nanoparticles (denoted as MS@Cu MNs), which enables efficient diabetic wound healing via a synergistic multimechanism strategy. Leveraging the unique enzyme‐mimetic activity of copper nanoclusters (CuNCs) and the angiogenic properties of magnesium silicate nanoparticles (MS NPs), the engineered MS@Cu nanocomposites demonstrate: 1) broad‐spectrum antibacterial efficacy (sterilization rate >99.9%), 2) microenvironment regulation via simultaneous hypoxia mitigation, ROS scavenging, and angiogenesis promotion, and 3) enhanced fibroblast proliferation and migration through PI3K‐AKT signaling pathway activation. The MN system using γ‐polyglutamic acid (γPGA) as a matrix exhibits both superior mechanical strength and excellent biodegradability. In vivo studies demonstrated accelerated closure of infected diabetic wounds in animal models, with histological analysis revealing robust mature collagen deposition and tissue regeneration. This study develops an integrated strategy for chronic diabetic wound management, combining potentiated antibacterial activity with targeted microenvironment remodeling.

## Graphical abstract

A microneedle dressing incorporating copper nanocluster‐decorated magnesium silicate nanoparticles has been fabricated for treating infected diabetic wounds through initial antibacterial effects, subsequent microenvironment regulation via simultaneous hypoxia mitigation, reactive oxygen species scavenging, and angiogenesis promotion, and fibroblast proliferation and migration promotion through PI3K‐AKT signaling pathway activation.

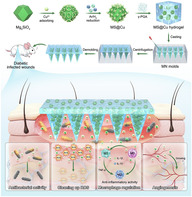

## 1 Introduction

Diabetic dermopathy is one of the most prevalent complications of diabetes mellitus, with an incidence rate as high as 16.8%.^[^ ^1^ ^]^ Both hyperglycemia and bacterial infections disrupt the wound microenvironment, leading to localized hypoxia, accumulation of reactive oxygen species (ROS), and diminished expression of growth factors.^[^ ^2^ ^]^ These pathophysiological alterations collectively result in inflammatory dysregulation, impaired angiogenesis, and reduced collagen production.^[^ ^2^ ^]^ The synergistic interplay of these pathophysiological processes drives the development of chronic nonhealing wounds, which in severe cases can culminate in limb amputation or mortality.^[^ ^3^ ^]^ Effective therapeutic strategies for chronic diabetic wounds necessitate a comprehensive multimodal approach integrating antimicrobial intervention, pro‐angiogenic stimulation, and anti‐inflammatory modulation to target the underlying pathophysiology. Critically, microbial infection remains a pivotal challenge, demanding biomaterials with dual‐functional capability, combining potent antimicrobial efficacy with tissue‐regenerative properties. While conventional therapies like systemic antibiotics and topical antiseptics demonstrate partial efficacy, their clinical utility is increasingly constrained by two pivotal factors: 1) the emergence of multidrug‐resistant bacterial strains and 2) the suboptimal wound microenvironment characterized by hypoxia, excessive proteolytic activity, and dysregulated immune responses that hinder conventional antimicrobial mechanisms.^[^ ^4^, ^5^ ^]^ These limitations underscore the urgent need for next‐generation therapeutic platforms integrating microenvironmental modulation with efficient antimicrobial effects.

Antimicrobial materials initially originated from metallic elements such as copper (Cu), silver (Ag), gold (Au), and zinc (Zn), which dominated infection control prior to the advent of antibiotics.^[^ ^6^ ^]^ However, the widespread clinical adoption of antibiotics led to the diminished utilization of metal‐ion‐based antimicrobial strategies. This trend has recently reversed due to the global crisis of antibiotic resistance and the emergence of multidrug‐resistant pathogens, reigniting interest in metal‐derived antimicrobial agents. Metal‐based materials exist in various forms, including single‐atom nanozymes, nanoclusters, nanoparticles, and metal‐ion‐doped scaffolds or coatings.^[^ ^7^ ^]^ Among these different forms, metal nanoclusters, sized between 2 and 5 nm and composed of dozens to hundreds of atoms, are gaining increasing attention due to their higher activity than nanoparticles and better stability than single atoms. For example, compared to gold nanoparticles, gold nanoclusters have better in vivo stability, absorption, and lower toxicity.^[^ ^8^ ^]^ Notably, copper nanoclusters (CuNCs) have emerged as a preeminent candidate in biomedical applications, primarily due to their optimal equilibrium between antibacterial efficacy and biocompatibility.^[^ ^9^ ^]^ Mechanistically, CuNCs exhibit superior antimicrobial potency compared to zinc and gold, as evidenced by their ability to achieve a 99.9% bacterial mortality rate at low concentrations.^[^ ^10^ ^]^ The strong antibacterial effects of CuNCs arise from their ability to destroy bacterial wall structures.^[^ ^11^ ^]^ Moreover, they can modulate the glutathione/glutathione disulfide (GSH/GSSG) ratio by inhibiting glutathione reductase activity, triggering a burst of ROS, and eventually causing bacterial death.^[^ ^11^ ^]^ In addition, copper‐based nanomaterials can mimic multiple enzymes (e.g., peroxidase (POD) and oxidase (OXD)), enhancing antibacterial effects through various mechanisms and effectively combating drug‐resistant bacteria.^[^ ^12^ ^]^ Unlike silver, copper can be metabolized well, and excess copper in the body can be easily excreted.^[^ ^13^ ^]^ Critically, copper's advantages extend beyond biological performance: its natural abundance, cost‐effectiveness, and established industrial infrastructure make it uniquely suited for scalable clinical translation.^[^ ^14^ ^]^

Copper, as an essential trace element in humans, plays a critical role in wound healing by modulating the synthesis and functional expression of extracellular matrix (ECM) components, including fibrinogen, collagen, and integrins.^[^ ^15^ ^]^ Besides, the latest research shows that copper has multiple biological activities in different tissue repair processes. For example, copper ions can activate dynamin‐related protein 1 (Drp1), thereby promoting mitochondrial mitophagy in osteoblasts.^[^ ^16^ ^]^ In another study, adding copper can activate the copper transporter ATP7A, thereby inhibiting autophagy and increasing the expression of VEGFR2.^[^ ^17^ ^]^ Furthermore, copper‐based nanomaterials can optimize the wound microenvironment by alleviating hypoxia, regulating local inflammatory responses, reducing inflammation around the wound, and improving the wound environment, further promoting the healing process.^[^ ^18^ ^]^

**Scheme 1:**
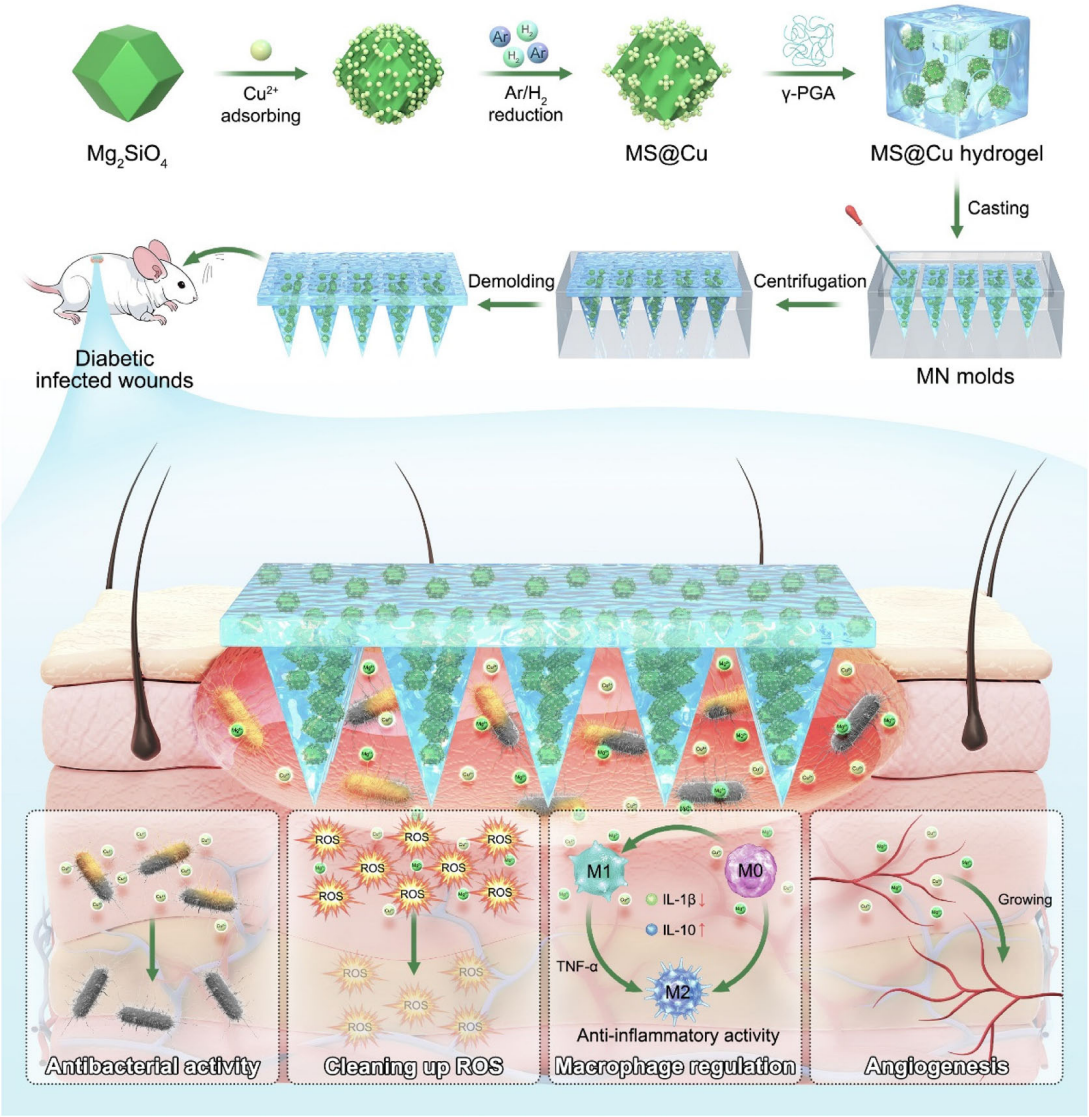
Schematic illustration of preparation and mechanism of the MS@Cu MNs for treating infected diabetic wounds.

Although copper ions can promote VEGF expression and angiogenesis,^[^ ^19^ ^]^ this effect is limited by the low copper concentration available in practical applications due to severe cytotoxicity induced at high copper concentrations. Therefore, it is necessary to incorporate nontoxic active substances that promote angiogenesis in the wound healing composites. Magnesium ions and silicate ions have excellent bioactivities to promote blood vessels and tissue regeneration.^[^ ^20^, ^21^ ^]^ In addition, silicate ions can enhance cell proliferation and migration by activating the integrin α 2 (ITGA2) gene in fibroblasts, simulating the microenvironment of scar‐free healing.^[^ ^22^ ^]^ Therefore, we chose magnesium silicate nanomaterials as carriers for CuNCs. The silicon oxygen dangling bonds on the surface of magnesium silicate nanoparticles (MS NPs) can be used to anchor copper ions to obtain copper ion‐absorbed MS NPs. By sintering the MS NPs absorbed with copper ions in a reducing atmosphere, CuNCs‐loaded MS NPs (MS@Cu NPs) were prepared. Finally, the MS@Cu NPs were added to the γ‐polyglutamic acid (γPGA) matrix to obtain MS@Cu microneedles (MNs) through a solvent casting method. γPGA is a natural biopolymer with high biocompatibility, degradability, hydrophilicity, and high mechanical properties, making it an ideal MN material.^[^ ^23^ ^]^ MNs have emerged as a promising platform for transdermal antimicrobial drug delivery, enabling precise local administration of therapeutic agents in a noninvasive manner while maintaining high patient compliance.^[^ ^23^, ^24^ ^]^ The transdermal effect of MNs can be used to deliver bioactive MS@Cu NPs to the wound sites to play the biological effects of clearing ROS of copper clusters and promoting vascular regeneration and epithelial cell regeneration of magnesium silicate, so as to better remold the traumatic microenvironment and repair diabetic skin damage (**Scheme** **1**).

## 2 Results and Discussion

### 2.1 Preparation and Characterization of CuNC@MS NPs

**Figure 1:**
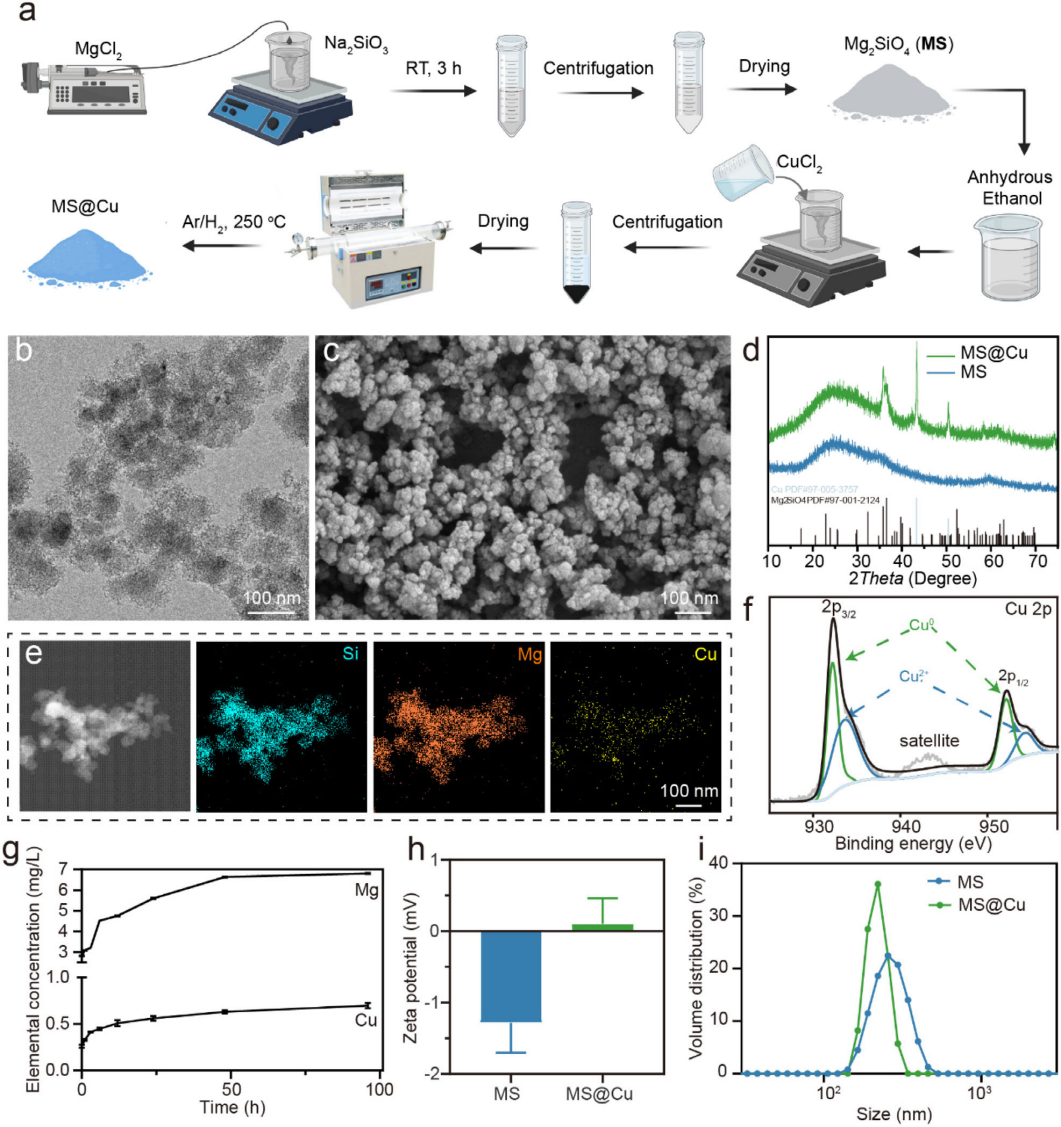
Material characterization of MS@Cu. a) Schematic diagram of material synthesis. b) TEM images and electron diffraction. c) SEM images, d) XRD analysis. e) Element mapping images. f) XPS analysis of MS@Cu. g) ICP analysis of ion release of MS@Cu in deionized H_2_O at pH 5.5. h) Zeta potential and i) Particle size distribution analysis. The data were presented as mean±SD.

The magnesium silicate nanoparticles (MS NPs) were prepared using a coprecipitation reaction. The copper nanoclusters (CuNCs) loaded MS NPs (MS@Cu) were prepared by sintering MS NPs that adsorbed copper ions (Cu^2+^) via a solid‐phase reduction reaction under the Ar/H_2_ atmosphere (**Figure** **1**a). Specifically, the magnesium silicate nanoparticles are formed via coprecipitation in aqueous solution using magnesium chloride and sodium silicate as precursors. The resulting nanoparticles are then dried, dispersed in an ethanolic solution of copper chloride, and subjected to copper ion adsorption, yielding copper‐ion‐adsorbed magnesium silicate nanoparticles. Subsequently, these particles are thermally treated under a protective reducing atmosphere (i.e., Ar/H_2_ gas mixture), where the surface copper ions are reduced to copper nanoclusters, ultimately producing the MS@Cu composite material. The morphology of MS@Cu was investigated using transmission electron microscope (TEM) and scanning electron microscope (SEM) (Figure 1b,c). The MS@Cu is composed of MS NPs smaller than 100 nm, with black dots of CuNCs dispersed on the surface of MS NPs. The elemental mapping performed using energy dispersive X‐ray spectrometry (EDS) shows that Mg, Si, and Cu elements are uniformly distributed on the MS nanoparticles (Figure 1e). The loading amount of copper element was determined to be 1.08 mg g^−1^ using the inductively coupled plasma‐atomic emission spectrometry method. The crystal phases of MS and MS@Cu were measured using X‐ray diffraction (XRD). The wide hump at ≈26.0° in the XRD pattern of the MS sample indicates the amorphous phase of magnesium silicate. After loading of CuNCs, the appearance of peaks at 35.5° and 59.8° indicates the existence of the orthorhombic phase of magnesium silicate (Mg_2_SiO_4_, PDF#97‐001‐2124), and the peak at ≈43.7° can be indexed to the cubic phase of copper (PDF#97‐005‐3757) (Figure 1d). To further investigate the chemical composition and electronic structure of MS@Cu, X‐ray photoelectron spectroscopy (XPS) was performed to determine the chemical valence of Cu. The XPS spectra of MS@Cu show the coexistence of all the compositional elements, including Mg, Si, O, and Cu elements, further confirming the successful loading of Cu on MS NPs (Figure S1, Supporting Information). The deconvoluted spectrum for the Cu 2p peaks located at 932.38 and 952.18 eV presents two doublets, demonstrating a main valence state of Cu^0^ together with Cu^2+^. The dominant peaks at 932.08 and 952.58 eV can be assigned to the 2p_3/2_ and 2p_1/2_ in the Cu 2p spectrum of Cu^0^, indicating that copper ions have been successfully reduced to metallic Cu. In addition, two minor peaks with the energy of 933.78 and 954.38 eV are assigned to 2p_3/2_ and 2p_1/2_ of Cu^2+^, indicating the existence of divalent copper element (Figure 1f). These divalent copper ions may be distributed on the surface of copper clusters to form bonding connections with magnesium silicate. These results demonstrate that the metallic Cu is successfully synthesized in our method, and it can be used as the active component for eliminating bacteria. Next, the release behaviors of Mg and Cu elements were investigated in the deionized water. The release amount of Mg and Cu ions rapidly increases in the first 12 h, then slowly increases between 12 and 48 h, and finally reaches a plateau after 48 h (Figure 1g). The Zeta potentials were measured to investigate the surface charge properties of MS and MS@Cu (Figure 1h). The results show that the Zeta potential changes from negative to positive after CuNCs loading, which may be due to the existence of partial divalent copper ions inside CuNCs. The hydrodynamic particle sizes of MS and MS@Cu were measured using dynamic light scattering (DLS). The particle size distribution curve shows monodispersity of both MS and MS@Cu samples, with the size of ≈220 nm for MS and ≈255 nm for MS@Cu (Figure 1i).

### 2.2 Preparation and Characterization of MS@Cu@MN

**Figure 2:**
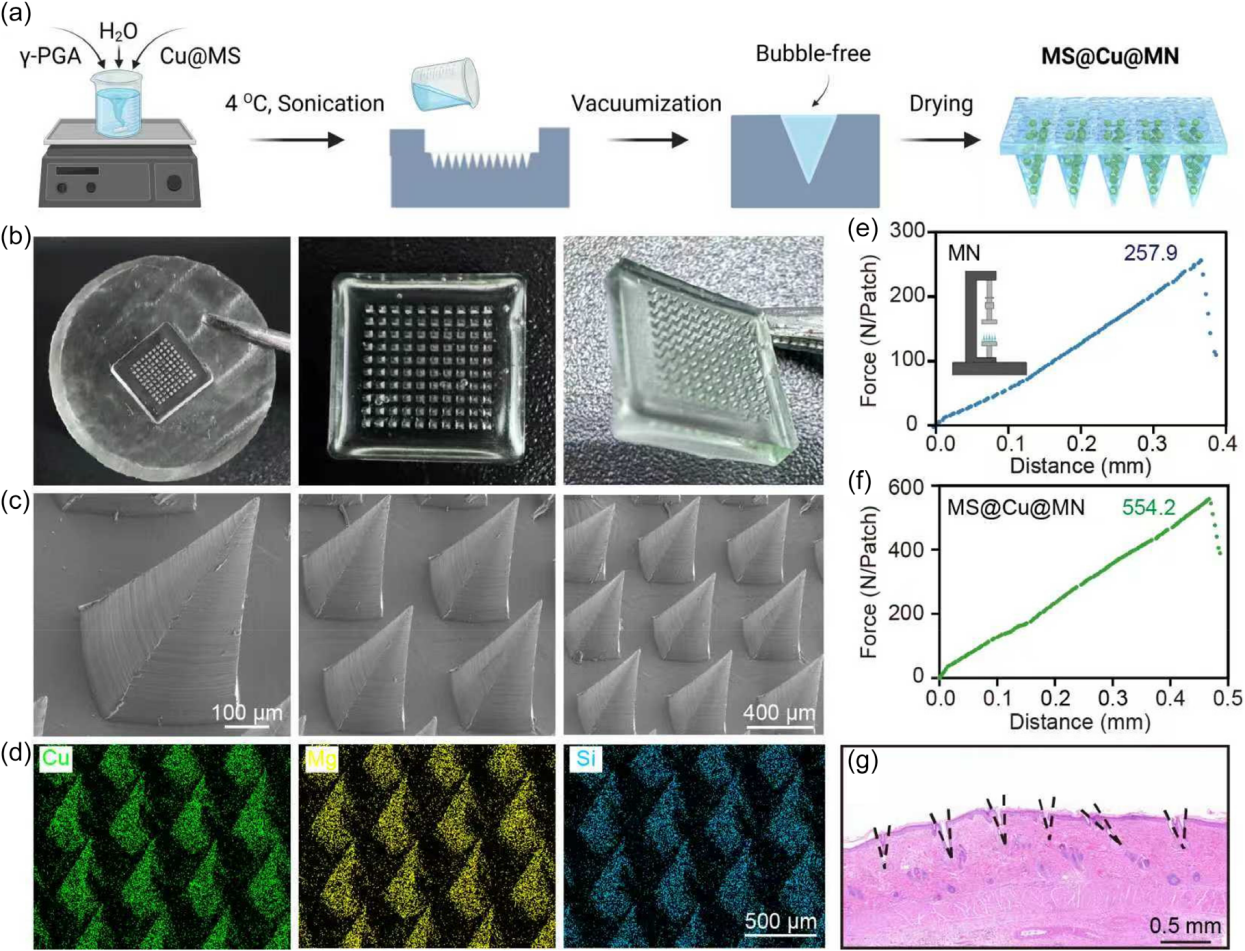
Preparation and Characterization of MS@Cu@MN. a) Schematic diagram of the MS@Cu@MN preparation process using a template replication method. b) Optical images of the MNs. c) SEM images. d) EDS images. e,f) Mechanical tests of the MN (e) and the MS@Cu@MN f). g) Tissue staining image showing the penetration of MS@Cu@MN.

γPGA, a natural biopolymer with high biocompatibility, degradability, hydrophilicity, and high mechanical properties, was selected as the MN matrix. The MN patch with a 10 × 10 needle array was prepared using a template replication method (**Figure** **2**a,b). The SEM images show that the MN patch has intact tips in a regular pyramid shape. This sharp shape is beneficial for transdermal delivery of active substances such as drugs using MNs (Figure 2c). EDS measurements show the uniform distribution of Cu, Mg, and Si elements within the MNs, indicating the successful fabrication of the MS@Cu loaded MN (MS@Cu@MN) patch (Figure 2d). The mechanical strength of the MN patch before and after loading MS@Cu was measured using a universal testing machine. The results show that the MN can withstand a pressure of 257.9 N (Figure 2e), and after incorporating MS@Cu nanoparticles, the pressure tolerance increased to 554.2 N (Figure 2f). The maximum strength of each needle is calculated to be 0.26 and 0.55 N for MS@Cu and MS@Cu@MN, which sufficiently surpasses the minimum force required for penetrating the stratum corneum (i.e., 0.045 N).^[^ ^21^ ^]^ Effective moisture‐management capability is crucial for wound dressings, as it maintains an appropriately moist microenvironment, facilitates exudate absorption during healing, and suppresses bacterial proliferation. Moisture‐uptake assays revealed that the needle tips of the MS@Cu@MN patch dissolved completely within 20 min, substantiating its superior moisture‐absorption performance and its capacity to sustain an ideal moist wound‐healing environment (Figure S2, Supporting Information). The tissue penetrating hematoxylin and eosin (HE) staining image shows that our MS@Cu@MN patch is capable of penetrating the rat skin dermis, which is consistent with the mechanical measurement results (Figure 2g). The HE‐stained tissue section demonstrates that the MNs on the MS@Cu@MN patch effectively penetrated the rat skin at multiple sites, confirming secure adhesion to the skin surface. These morphological findings are consistent with the mechanical strength data previously obtained (Figure 2g).

### 2.3 Biocompatibility and Bioactivity Characterization

**Figure 3:**
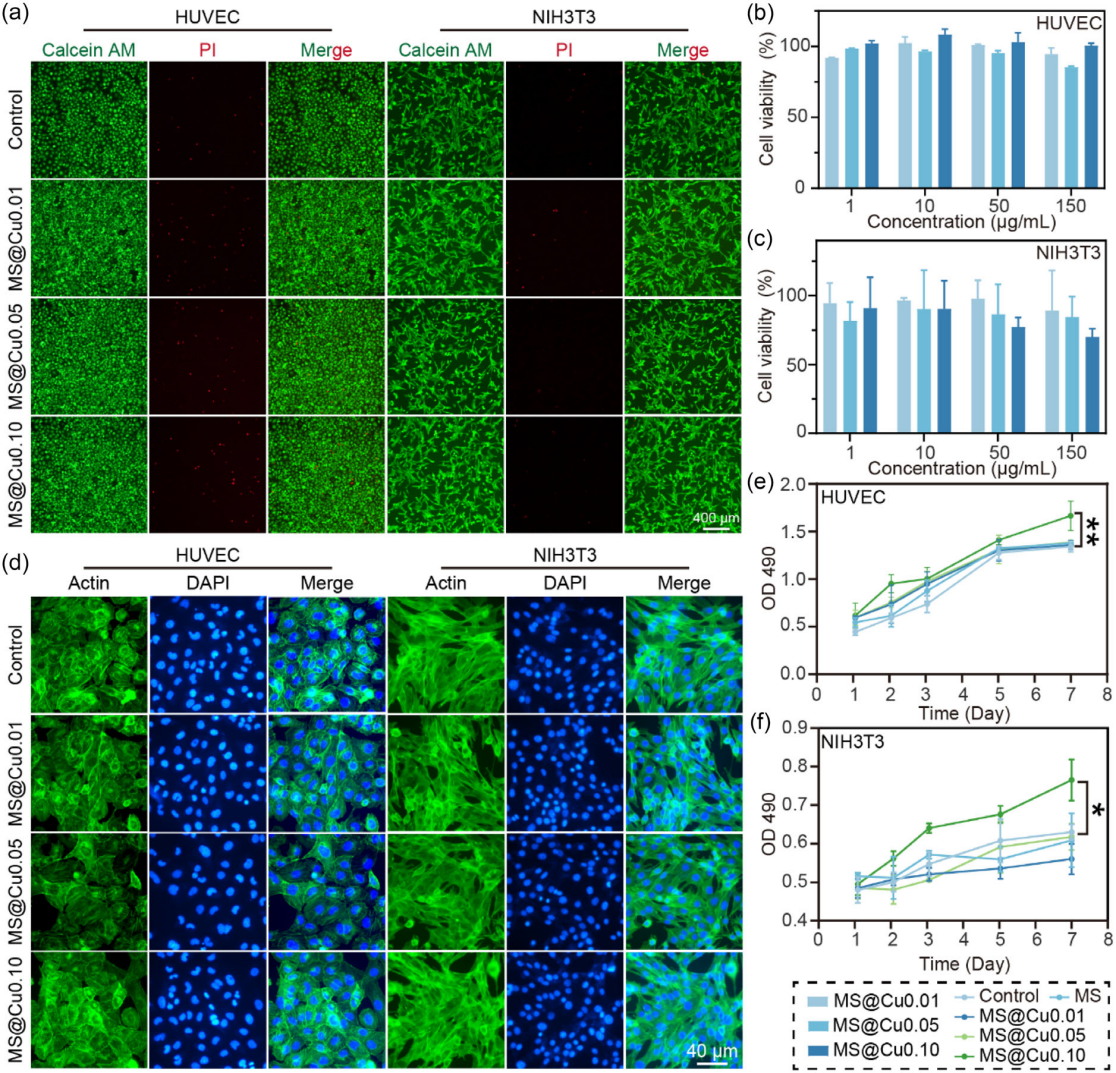
Biocompatibility assessment of MS@Cu samples with different copper content. a) Representative live/dead staining images of cells cultured with MS@Cu samples (10 μg mL^−1^) for 24 h. b,c) Cell viability following 24 h exposure to the materials (*n* = 3). d) Representative immunofluorescence images visualizing filamentous actin in cells cocultured with MS@Cu samples (10 μg mL^−1^) for 24 h. e) HUVEC cells proliferation assessed after treatment with MS@Cu samples (10 μg mL^−1^, *p* = 0.0043). f) NIH3T3 cells proliferation assessed after treatment with MS@Cu samples (10 μg mL^−1^, *p* = 0.0193). Data analysis was conducted using paired t‐tests. Statistical significance: *p < 0.05, **p < 0.01.

The cytotoxicities of MS samples loaded with different CuNCs amount (i.e., from 0.01 to 0.10 mmol) were characterized using human umbilical vein endothelial cells (HUVECs) and the rat fibroblast NIH/3T3 cell lines (**Figure** **3**a). When cocultured with HUVECs, cell viability remained nearly 100% at material concentrations up to 150 μg mL^−1^ across all samples with varying CuNCs content, indicating low cytotoxicity of the MS@Cu samples (Figure 3b). In contrast, MS@Cu induced a certain degree of cytotoxicity in NIH3T3 cells with increasing material concentration or increasing copper content in the MS@Cu (Figure 3c). Therefore, a low material concentration of 10 μg mL^−1^ was selected for subsequent cell proliferation experiments. Cytoskeletal structures, particularly filamentous proteins, provide essential mechanical support critical for maintaining cell shape and facilitating processes such as migration, including lamellipodia and filopodia formation. Consequently, their organization serves as a key indicator of cellular viability and migratory potential. To evaluate these properties following coculture with MS@Cu samples with different copper content, the cytoskeletal architecture of HUVECs and NIH/3T3 cells was characterized using immunostaining (Figure 3d). The results indicate that both HUVECs and NIH/3T3 cells maintained normal proliferation and morphology across all experimental groups. The cell immunofluorescence staining images clearly show the uniform cellular filamentous proteins and good spread state of cells in the blank and other material groups. Furthermore, the overall cellular architecture showed no significant changes compared to the control group, indicating the good biocompatibility of the MS@Cu0.10 materials. Cell proliferation assays indicate that the MS@Cu0.10 group significantly enhanced cell proliferation compared to the blank control and other material groups (Figure 3e,f). This pro‐proliferative effect likely involves the biological activity of copper ions gradually released from the MS@Cu sample over the 7‐day coculture period.

**Figure 4:**
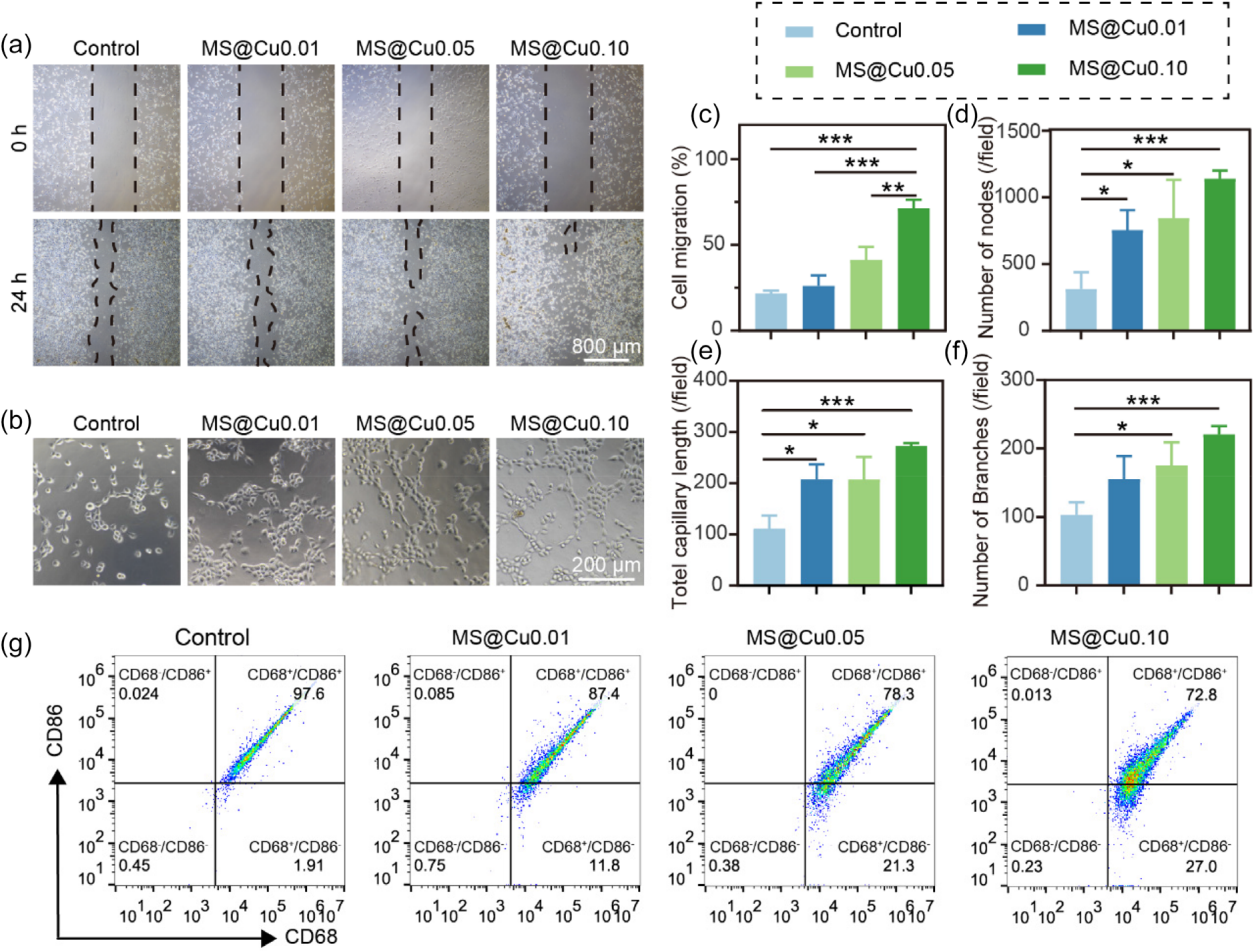
Pro‐angiogenic and pro‐migratory effects of MS@Cu (10 μg mL^−1^) on endothelial and fibroblast cells. a) Migration of NIH/3T3 fibroblasts assessed by scratch assay after 24 h exposure to MS@Cu. b) Angiogenic tube formation of HUVECs after 4 h treatment with MS@Cu. c) Quantification of cell migration rate (*n* = 3). d–f) Angiogenesis parameters: number of tubular junctions (d, *n* = 3), total capillary length (e, *n* = 3), and branch points (f, *n* = 3). g) Polarization of RAW264.7 macrophages treated with MS@Cu for 24 h, detected by the flow cytometry analysis. The data were presented as mean±SD. Data analysis was conducted using one‐way analysis of variance. Significance: *p < 0.05, **p < 0.01, and ***p < 0.001.

The biological functions of our MS@Cu samples, including the promotion effects for cell migration, angiogenesis, and polarization, were subsequently investigated using HUVECs and NIH/3T3 cells. The scratch assay was applied to investigate the migration ability of NIH/3T3 cells cultured with different groups. The results show that the migration rate of NIH/3T3 cells is directly proportional to the copper content in the material after coculture with materials for 24 h (**Figure** **4**a). The healing percentages for Control, MS@Cu0.01, MS@Cu0.05 and MS@Cu0.10 groups are determined to be 21 ± 2%, 26 ± 6%, 41 ± 7%, and 71 ± 5%, respectively (Figure 4c). This result proves that the Cu^2+^ ions released from MS@Cu samples can promote cell migration, which is consistent with the filamentous protein staining results. The quantitative analysis results show that the migration rate of NIH/3T3 cells in the MS@Cu0.10 group is 3.4‐fold higher than that in the control group. Furthermore, a tubule formation assay was conducted to evaluate the effects of the samples on the angiogenic potential of HUVECs (Figure 4b). The impact of different MS@Cu samples on the angiogenic potential of HUVECs was quantitatively evaluated by the number of ring‐like nodes, total capillary length, and the number of branches (Figure 4d–f). In contrast to the blank group, the number of circular nodes and branches and total capillary length were significantly enhanced upon the addition of MS@Cu samples with even low copper content. This significant enhancement of HUVECs’ angiogenesis by the MS@Cu material with low copper content is due to the bioactive effects of silicate and magnesium ions in the material. In particular, as the copper content increases, the pro‐angiogenic effect is also enhanced. This result implies that the copper also plays a positive role in promoting angiogenesis. The above results indicate that our nanomaterial can promote cell migration, angiogenesis, and possess anti‐inflammatory biological functions. The effects of MS@Cu samples on promoting macrophage polarization were further studied using RAW264.7 cells. Flow cytometry analysis demonstrates a dose‐dependent reduction in the proportion of RAW264.7 cells in the Q2 phase (G2/M phase) upon treatment with MS@Cu materials containing increasing copper concentrations (from 0.01 to 0.10 mmol), with the Q2 population decreasing from 96.7% (control) to 72.6% in the MS@Cu0.10 group (*p* < 0.05). This trend suggests that higher copper concentrations in the MS@Cu material can induce a dose‐dependent downregulation of M1‐polarized macrophages. A reduction in the promotion of inflammatory phenotypes is beneficial for tissue regeneration and repair. These results indicate that the MS@Cu has multiple biological functions of cell migration promotion, angiogenesis promotion, and macrophage modulation.

### 2.4 ROS Elimination and Antibacterial Properties

**Figure 5:**
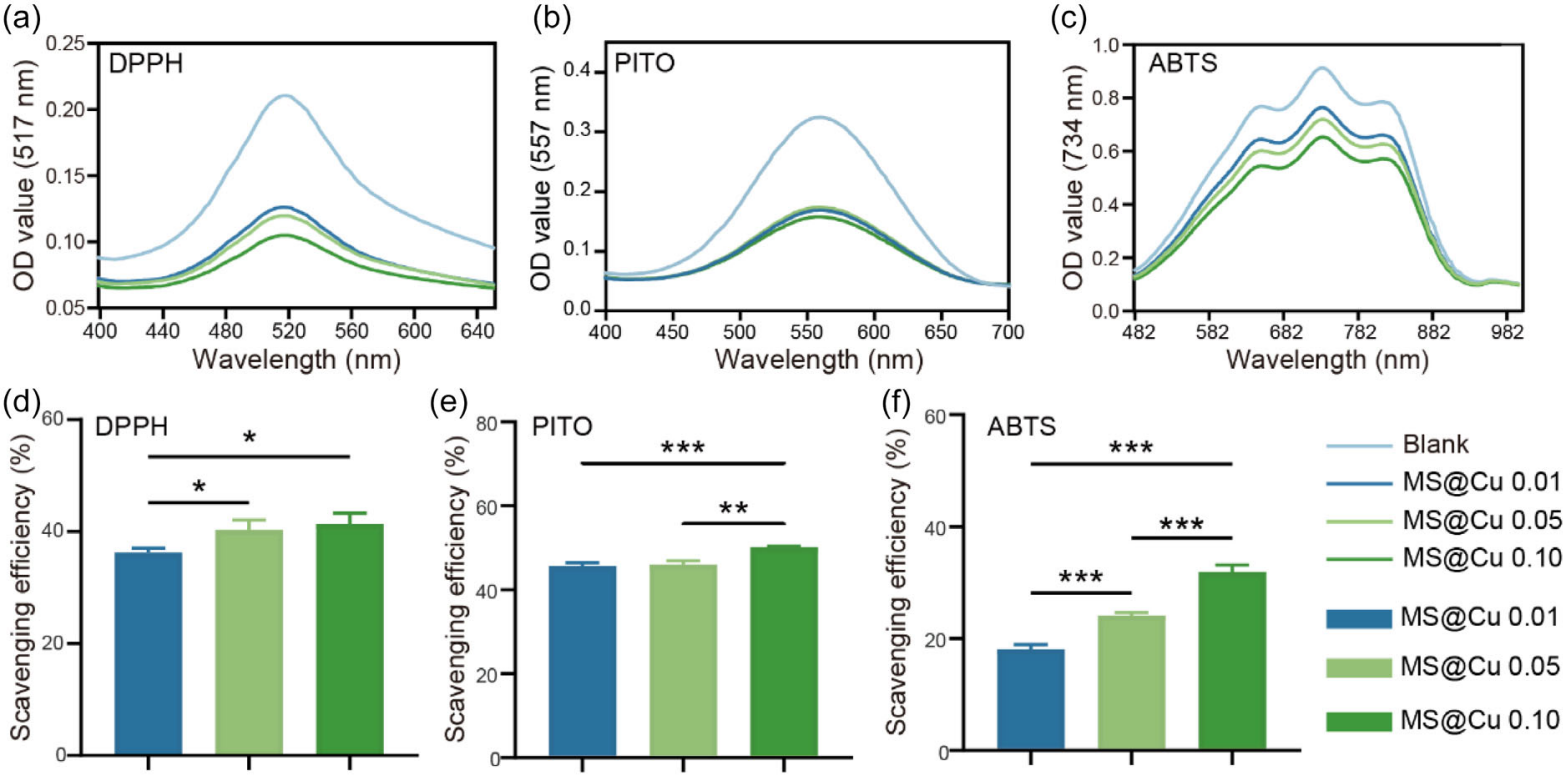
Free radical scavenging activity of MS@Cu samples. a–c) UV–visible absorption spectra measured after coculturing different MS@Cu samples (150 μg mL^−1^) with ABTS, DPPH, and PTIO radicals, respectively. d–f) Quantification of scavenging rates of different MS@Cu samples against different radicals (*n* = 3). The data were presented as mean±SD. Data analysis was conducted using one‐way analysis of variance. Significance levels: *p < 0.05, **p < 0.01, and *p < 0.001.

Excessive accumulation of ROS is the main characteristic of the inflammatory stage, and excessive ROS hinders cell viability and tissue regeneration. Therefore, the ability to clear free radicals is an important characteristic of skin repair dressings. The ability of our MS@Cu material to scavenge free radicals was evaluated by coculturing with typical free radical molecules, including nitrogen‐free radical substrates (e.g., ABTS and DPPH) and oxygen‐free radical substrates (e.g., PTIO•). The optimal concentration for ROS scavenging by MS@Cu0.10 was initially determined for all three radicals using the MS@Cu0.10 sample (Figure S3, Supporting Information). Scavenging efficiency by MS@Cu0.10 exhibited concentration dependence for each radical, reaching a maximum at 150 μg mL^−1^. Subsequently, the ROS scavenging capacities of MS@Cu samples with varying copper contents were evaluated at a fixed concentration of 150 μg mL^−1^. The results demonstrate that the MS@Cu0.10 sample exhibits superior scavenging effects against all three free radicals compared to MS@Cu0.01 and MS@Cu0.05 (**Figure** **5**a–c). The scavenging capacities of MS@Cu0.10 for ABTS, DPPH, and PTIO radicals reached 50.1% ± 0.3%, 41.4% ± 1.9%, and 31.9% ± 1.3%, respectively (Figure 5d–f). Given that both magnesium ions and silicate ions lack intrinsic free radical scavenging activity, the observed antioxidant effects originate exclusively from CuNCs immobilized on the MS nanoparticles. These collective findings demonstrate that MS@Cu exhibits potent and broad‐spectrum ROS elimination capabilities, thereby offering significant potential to mitigate ROS‐induced tissue damage and accelerate regenerative processes. Given the good ROS scavenging ability of the MS@Cu material, we further studied its intracellular ROS scavenging ability. The experimental results demonstrated that the intracellular ROS level in the MS@Cu0.10 + H_2_O_2_ cotreatment group was significantly lower than that in the H_2_O_2_‐only group, and was reduced to a level comparable to that of the Control group, indicating that MS@Cu0.10 effectively attenuated H_2_O_2_‐induced oxidative stress in NIH3T3 cells (Figure S4, Supporting Information).

**Figure 6:**
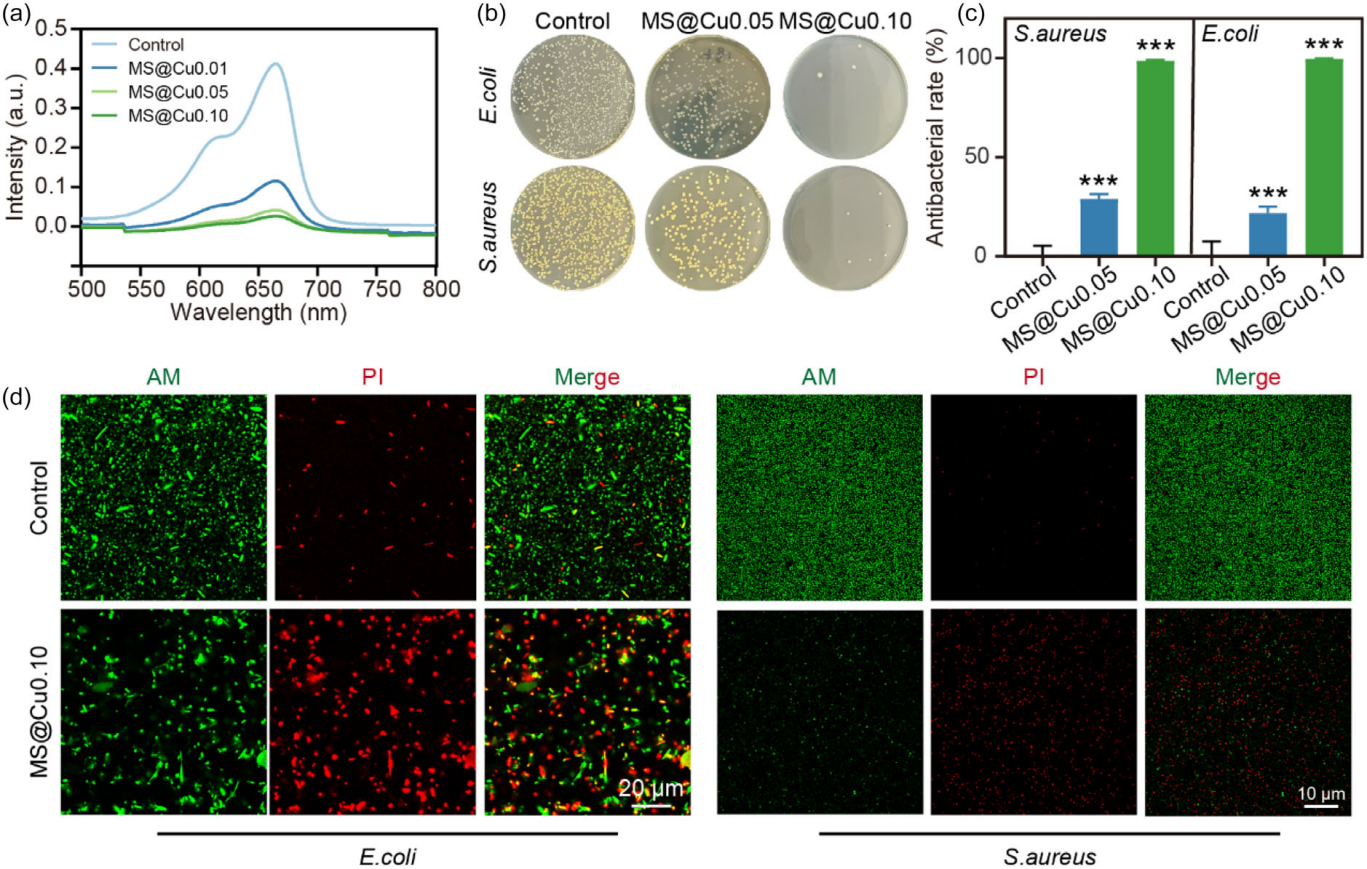
Antimicrobial‐related testing. a) Fenton‐like reactivity assays of MS@Cu materials at a concentration of 150 μg mL^−1^. b) Colony formation unit assays of both *S. aureus* and *E. coli* treated with different MS@Cu samples. c) Quantitative analysis of bacterial colonies *n* = 3). d) Live/dead staining images of both *S. aureus* and *E. coli* bacteria after exposure to MS@Cu0.10 NPs. The data were presented as mean±SD. Data analysis was conducted using one‐way analysis of variance. Significance levels: *p < 0.05, **p < 0.01, and *p < 0.001.

Diabetic skin wounds exhibit high susceptibility to bacterial infections, which significantly impede the healing process. Consequently, effective eradication of pathogenic bacteria constitutes an essential requirement for advanced wound dressings. During the initial ROS scavenging process, zero‐valent copper atoms in the MS@Cu NPs undergo oxidation to monovalent (Cu^+^) or divalent (Cu^2+^) states via electron transfer. These bioactive copper ions (Cu^2+^/Cu^+^) will subsequently be released from MS@Cu NPs to exert antibacterial activity by disrupting bacterial DNA, lipids, and proteins through a Fenton‐like reaction. Fenton‐like reactivity assays confirmed the redox activity of MS@Cu NPs, with oxidation efficiencies of 72%, 90%, and 94% for MS@Cu0.01, MS@Cu0.05, and MS@Cu0.10, respectively (**Figure** **6**a). The antibacterial activities of our MS@Cu samples were evaluated using both Gram‐positive and Gram‐negative bacteria. The bacterial colonies counting results demonstrate that the MS@Cu0.10 group has high sterilization efficiency for both *S. aureus* (i.e., 99.5%) and *E. coli* (i.e., 99.8%) compared to the MS@Cu0.05 group (Figure 6b,c). The excellent antibacterial efficacy of MS@Cu0.10 is due to the high CuNCs content loaded on the MS matrix. It is reported that the ultrasmall size (i.e., 1–5 nm) and high surface‐to‐volume ratio of CuNCs enhance their antimicrobial efficacy through facilitating deep penetration into bacterial membranes and biofilm matrices.^[^ ^11^ ^]^ The live/dead bacterial viability staining was assessed using a membrane‐permeable green fluorescent dye (i.e., Calcein AM) for live bacteria and a red fluorescent nucleic acid stain (i.e., propidium iodide, PI) for dead bacteria (Figure 6d). Confocal laser scanning microscopy images show the distinct spatial segregation of green (live) and red (dead) fluorescence signals within bacterial colonies. It was observed that when cocultured with the MS@Cu0.10 sample, the live bacterial population (i.e., green fluorescence) significantly decreased, while the dead bacterial population (e.g., red fluorescence) significantly increased for both *S. aureus* and *E. coli*, aligning with prior studies of colony‐forming unit assays, which further confirmed a >99% reduction in culturable bacteria.

### 2.5 In Vivo Wound Healing of Infected Diabetic Skin Defects

**Figure 7:**
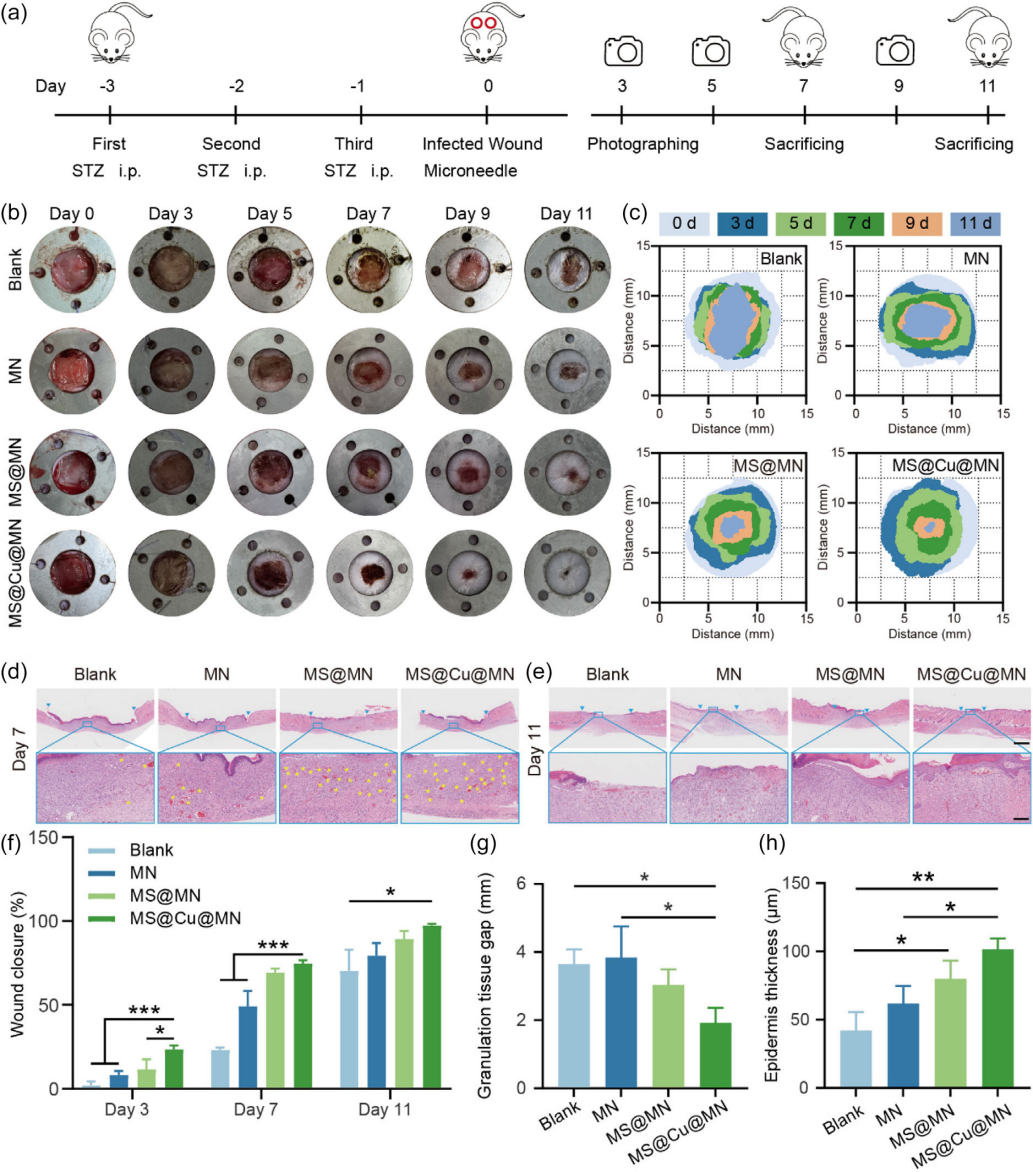
In vivo evaluation of wound healing in a rat skin injury model. a) Experimental design schematic. b) Representative wound images from postoperative days 0–11. c) Quantitative analysis of wound closure kinetics. d,e) HE‐stained sections at day 7 d) and 11 (e, neovessels denoted by yellow asterisks). f–h) Statistical charts of wound closure (f), granulation tissue gap (g), and epidermis thickness (h) of different groups (*n* = 3). The data were presented as mean±SD. Data analysis was conducted using one‐way analysis of variance. Significance levels: *p < 0.05, **p < 0.01, and *p < 0.001.

The synergistic antibacterial efficacy and pro‐regenerative functions for cell migration, angiogenesis, and polarization of our MS@Cu imply that the material can have a good therapeutic effect on wound healing when incorporated in MNs. Full‐thickness defects were constructed on the back skin of diabetic rats as a wound model to evaluate the in vivo wound healing ability of the MS@Cu@MN (**Figure** **7**a). An annular stainless steel ring (inner diameter: 10 mm; outer diameter: 20 mm; thickness: 1 mm) was surgically implanted at the wound site. The ring's internal circumference was precisely aligned with the epidermal defect to mitigate tissue contraction during healing. Then, standardized inocula of *S. aureus* (1 × 10^9^ CFU mL^−1^, 10 μL) were intradermally administered into the wound beds. At 24 h post‐inoculation, prominent inflammatory reactions characterized by perilesional erythema and edema were observed, with copious purulent exudate confirming active infection (Figure 7b). Animals were subsequently randomized into five groups: untreated control (Blank) and treated with different materials, including MN patch alone, MNs incorporating magnesium silicate (MS@MN), and MNs incorporating MS@Cu (MS@Cu@MN). The wound closure progression was documented photographically on days 0, 3, 5, 7, 9, and 11 to quantitatively evaluate healing dynamics. Longitudinal analysis revealed a time‐dependent reduction in infected wound surface area across all experimental groups. However, the rate of wound area reduction varied significantly between groups. For example, at postoperative day 11, the mean lesion size in the MS@Cu@MN group decreased to 2.1 ± 0.8 mm^2^, whereas the control group showed only a reduction to 20.8 ± 10.9 mm^2^ (Figure 7c). Furthermore, differential pathological manifestations were observed among the experimental groups. On postoperative day 7, purulent crust formation was evident on wounds in both the Blank and MN groups. In contrast, the MS@Cu@MN group exhibited robust granulation tissue formation throughout the wound bed, including peripheral and central regions (Figure 7b). Notably, by postoperative day 11, wounds in the MS@Cu@MN group exhibited near‐complete reepithelialization with only residual erythema. Quantitatively, wound closure rates were determined to be 70.2 ± 12.6%, 79.4 ± 7.6%, 89.3 ± 4.7% and 97.4 ± 0.9% for the Blank, MN, MS@MN, and MS@Cu@MN groups (Figure 7e). These in vivo results demonstrate that the MS@Cu@MN group achieved accelerated skin regeneration, with a closure rate 1.4‐fold higher than the Blank group. Tissue repopulation within the wound bed, granulation tissue formation, and the reepithelialization process were assessed via HE staining on postoperative days 3 and 7. On postoperative days 3 and 7, all groups exhibited irregular keratinocyte migration and incomplete reepithelialization at the wound margins, suggesting that the skin tissue had not yet fully regenerated.

By postoperative day 11, residual wound gap measurements revealed significant differences across groups, with the values of 3.64 ± 0.44, 3.84 ± 0.91, 3.03 ± 0.46, and 1.92 ± 0.44 mm for the Blank, MN, MS@MN, and MS@Cu@MN groups. The epidermis thickness of the Blank, MN, MS@MN, and MS@Cu@MN groups on Day 11 was determined to be 42.12 ± 13.49, 61.77 ± 12.86, 79.92 ± 13.3, and 101.27 ± 8.14 μm. The MS@Cu@MN group demonstrated the most favorable healing parameters, exhibiting both the narrowest residual wound gap and greatest epidermal thickness among all cohorts. This synergistic enhancement suggests superior reepithelialization capacity. The process of reepithelialization during skin repair is often accompanied by neovascularization. The newly‐formed blood vessels are indicated using a yellow star in the HE images. Significantly greater neovascularization was observed in the MS@Cu@MN group, indicating that the MS@Cu material has excellent pro‐angiogenic capability. These newly‐formed blood vessels facilitate local blood circulation and oxygen and growth factors transport, thereby substantially accelerating the regenerative process.

**Figure 8:**
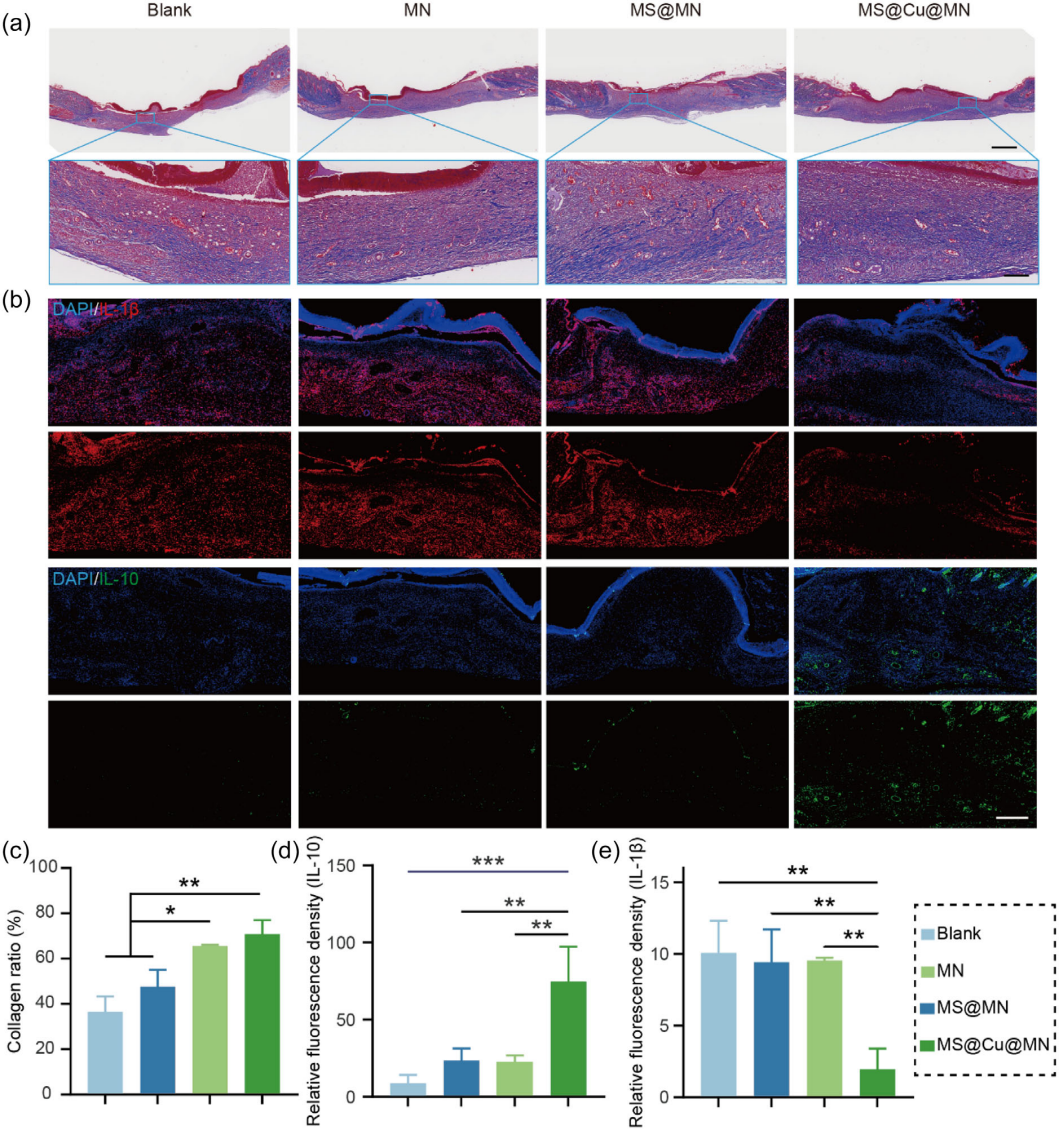
Histological and immunohistochemical assessment of rat wound healing. a) Representative Masson's trichrome‐stained sections (blue: collagen deposition). b) Fluorescence immunohistochemistry of IL‐1β and IL‐10. c) Quantification of collagen density from Masson's staining (*n* = 3). d,e) Statistical analysis of fluorescence staining of inflammatory cytokines IL‐1β (d, *n* = 3) and IL‐10 (e, *n* = 3). The data were presented as mean±SD. Data analysis was conducted using one‐way analysis of variance. Significance levels: *p < 0.05, **p < 0.01, and *p < 0.001.

Collagen deposition critically mediates tissue remodeling by facilitating ECM reconstitution and structural reinforcement of regenerating tissue, thereby driving the final healing phase. The extent of collagen deposition across experimental groups was quantitatively assessed via Masson's trichrome staining on postoperative day 7 (**Figure** **8**c). Quantitative analysis of Masson's trichrome‐stained sections revealed significantly greater collagen deposition in the MS@Cu@MN group compared to other groups, evidenced by greater relative staining intensity (Figure 8a). The collagen density was calculated to be 36.5 ± 6.9%, 47.6 ± 7.4%, 65.5 ± 0.6% and 70.8 ± 6.2% for the Blank, MN, MS@MN, and MS@Cu@MN groups. Masson's trichrome staining results revealed that the MS@Cu@MN group exhibited the highest collagen content on day 7, highlighting the material's potential to enhance wound healing and repair. This finding provides a plausible explanation for the accelerated skin repair observed in the MN‐MS@Cu group compared to other groups. In terms of anti‐inflammatory effects, the levels of two key inflammatory cytokines, IL‐1β and IL‐10, were measured. A reduction in IL‐1β expression suggests a gradual attenuation of the inflammatory response, while elevated IL‐10 levels indicate an environment conducive to tissue repair, as IL‐10 mitigates inflammation by suppressing pro‐inflammatory cytokine production and release (Figure 8b). Quantitative analysis of immunofluorescence staining results confirmed significantly downregulated IL‐1β expression and upregulated IL‐10 expression in the MN‐MS@Cu group compared to other groups (Figure 8d,e). These findings demonstrate the material's robust anti‐inflammatory properties. The anti‐inflammatory effect is primarily attributed to the scavenging of ROS by the copper clusters and the modulation of inflammatory cytokines by copper ions. The release of copper ions occurs through the oxidation of copper clusters within the MS@Cu NPs upon interaction with ROS in the inflammatory microenvironment. Notably, the generated copper ions exhibit intrinsic antibacterial properties by disrupting bacterial membranes and mediating Fenton‐like reactions (Figure 6). As a result, the MN‐MS@Cu system exerts simultaneous anti‐inflammatory and antibacterial effects. Overall, the animal experimental data underscore the exceptional skin repair efficacy and anti‐inflammatory capabilities of MS@Cu nanomaterials, offering promising insights for clinical applications in skin repair.

**Figure 9:**
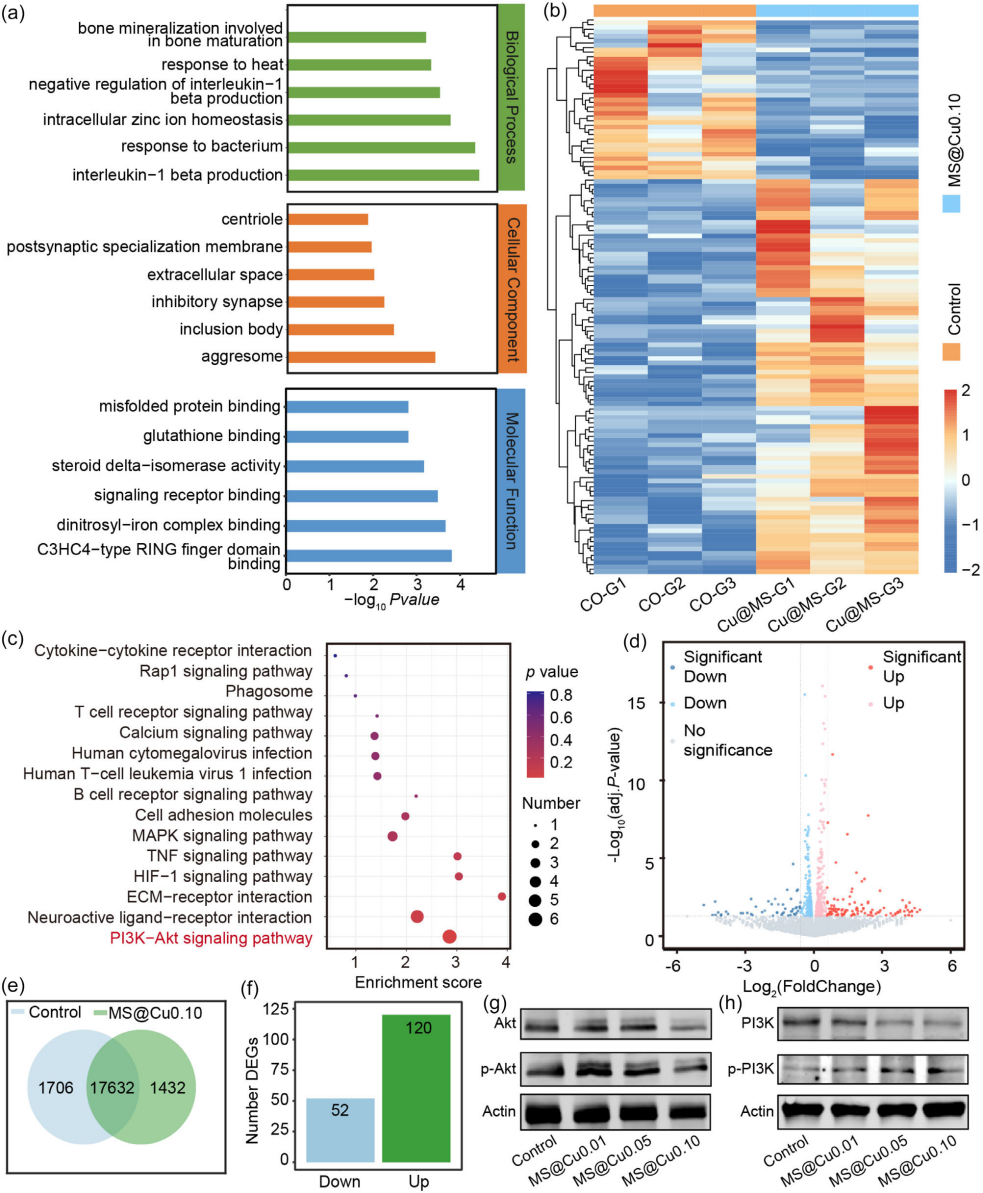
Analysis of gene transcriptome sequencing of NIH3T3 cells treated with MS@Cu0.10. a) GO functional analysis of the DEGs. b) Differential gene clustering diagram, where red indicates relatively high expression of protein‐coding genes and blue indicates relatively low expression of protein‐coding genes. c) KEGG enrichment bubble chart, where the horizontal axis Enrichment Score represents the enrichment value, and larger bubbles indicate a greater number of differentially expressed protein‐coding genes. d) Differential expression volcano diagram. e) Venn diagram comparing the treated group and the control group. f) The number of upregulated and downregulated genes. g,h) Western blot validation of the PI3K‐AKT signaling pathway.

To elucidate the anti‐inflammatory mechanisms and skin regeneration pathways mediated by MS@Cu nanomaterials, the gene transcriptome sequencing of NIH3T3 cells treated with MS@Cu0.10 nanomaterials was measured and analyzed (**Figure** **9**). RNA sequencing analysis identified 1432 differentially expressed genes (DEGs) in MS@Cu0.10‐treated NIH3T3 cells versus controls, representing 8.2% of total transcribed genes (*n* = 19 064). Among these, 172 genes showed statistically significant differential expression, with 120 upregulated and 52 downregulated transcripts (Figure 9a, d–f). Quantitative analysis of gene distribution shows that these different genes are related to the biofunctions, including cell proliferation, angiogenesis, and anti‐inflammatory effects. The effects of MS@Cu0.10 on the biological process, cellular component, and molecular function in NIH3T3 cells can be obtained using the gene ontology (GO) analysis (Figure 9a, b). The results show that the DEGs in the MS@Cu‐treated group were enriched in the immunomodulatory, inflammation‐related pathways, and antibacterial properties. Among the enriched pathways, cytokine‐cytokine receptor interaction and T‐cell receptor signaling were significantly overrepresented. This suggests that MS@Cu0.10 treatment could exert a regulatory effect on immune cell function. Furthermore, the PI3K‐Akt signaling pathway (*P* = 0.05) demonstrated significant enrichment (enrichment fraction = 4), indicating its potential role in regulating cell survival and metabolism (Figure 9c). The analysis of 120 DEGs shows that up‐regulated genes were dominant (such as Akt‐related genes), while the downregulated genes were mainly enriched in the MAPK signaling pathway and HIF‐1 signaling pathway (*P* = 0.6). This result is consistent with the trend of inflammatory pathway activation and metabolic pathway inhibition in the enrichment analysis (Figure 9e).

The PI3K‐AKT signaling pathway regulates diverse cellular functions—including metabolism, proliferation, survival, growth, and angiogenesis—that are critically involved in skin repair. To investigate its role, NIH/3T3 fibroblasts were cocultured for 24 h with MS@Cu nanomaterials containing varying copper loadings. Cells were subsequently harvested for western blot analysis to quantify changes in key PI3K‐AKT pathway proteins. Western blot analysis revealed increased phosphorylation ratios of both PI3K (*p*‐PI3K/PI3K) and AKT (*p*‐AKT/AKT) following material treatment (Figure 9g, h). These phosphorylation events exhibited dose‐dependent enhancement with elevated copper content, indicating progressive pathway activation. Collectively, these results demonstrate that MS@Cu nanomaterials promote cell proliferation and angiogenesis through PI3K‐AKT pathway activation—molecularly validating prior KEGG enrichment predictions.

## 3 Conclusions

This study introduces copper nanocluster‐decorated magnesium silicate nanoparticles (MS@Cu) as a multifunctional nanotherapy platform that overcomes the efficacy‐toxicity trade‐off inherent to copper‐based diabetic wound therapies. By integrating transdermal delivery of Mg^2+^/SiO_3_^2−^ ions and copper nanoclusters (CuNCs) within a stratified γ‐polyglutamic acid (γPGA) MN matrix, MS@Cu achieves spatiotemporal coordination of antibacterial action and immune modulation, critical for addressing the complex pathophysiology of diabetic wounds. During the initial repair phase, CuNCs effectively scavenge excess ROS, undergoing oxidation to release Cu^2+^ ions that regulate the wound microenvironment through anti‐inflammatory, antibacterial, and pro‐angiogenic mechanisms. In vivo experiments demonstrated accelerated diabetic wound closure with histological evidence of mature collagen deposition and tissue regeneration. Transcriptomics confirmed PI3K‐AKT pathway activation as the primary mechanism underlying MS@Cu's therapeutic efficacy.

## 4 Experimental Section

4.1

4.1.1

##### Synthesis of MS@Cu

A 0.6 mol L^−1^ MgCl_2_ solution (5 mL) was prepared, and a 0.06 mol L^−1^ Na_2_SiO_3_ solution (50 mL) was prepared. The MgCl_2_ solution was added dropwise to the Na_2_SiO_3_ solution at a rate of 2.5 mL h^−1^. After the addition, the mixture was stirred at room temperature for 3 h. Finally, the mixture was centrifuged, the supernatant was discarded, and the precipitate was rinsed three times with deionized water and anhydrous ethanol, then dried and stored for later use. 20 mg of the dried MS was dissolved in 14 mL of anhydrous ethanol, followed by the addition of 0.01, 0.05, and 0.10 mmol of CuCl_2_, and the solution was stirred for 24 h. The dried material was placed in a tube furnace and sintered in an argon–hydrogen mixed gas atmosphere at 250 °C for 2 h to obtain the MS@Cu sample.

##### Preparation of MN Patches

A MN mold composed of a 10 × 10 array (Micropoint Technologies Pet. Ltd., Singapore) was used, producing MN patches with tip dimensions of 310 × 310 × 680 μm (width × length × height). 0.32 g of γ‐PGA (molecular weight 800 000, Shanghai YuanYe, China) was dissolved in 1 mL of ddH_2_O containing 2.5 μg mL^−1^ of MS@Cu0.1. After dissolution, the solution was sonicated in ice water at 4 °C for 20 min. Subsequently, the resulting gel‐like liquid was filled into the MN mold and placed under vacuum for 5 min. This vacuum step was repeated until no bubbles were observed in the liquid of the MN mold. The filled mold was dried in a 40 °C oven for 6 h. The dried MN patches were stored under vacuum for future use.

##### Material Characterization

The following equipment was used for material characterization: Transmission Electron Microscope (TEM; JEOL JEM‐F200, Japan), Scanning Electron Microscope (SEM; ZEISS Gemini SEM 300, Germany), ICP‐OES/MS (Agilent 720 ES(OES), USA), Nanoparticle Size and Zeta Potential Analyzer (DLS; Malvern Zetasizer Nano ZS90, UK), X‐ray Photoelectron Spectrometer (XPS; Thermo Scientific K‐Alpha, USA), X‐ray Diffractometer (XRD; Rigaku SmartLab SE, Japan), and Pressure Tester (Shanghai Hengyu HY‐940FS, China).

##### MS@Cu In Vitro Antioxidant Experiment

1) DPPH (2,2‐diphenyl‐1‐picrylhydrazyl, Aladdin, Shanghai, China): A 1 μg mL^−1^ DPPH solution was prepared in anhydrous ethanol, diluted 20 times before use. A volume ratio of 1:1 between DPPH and MS@Cu solution was maintained during the reaction, resulting in a final MS@Cu concentration of 150 μg mL^−1^. The reaction was incubated on a shaking incubator at 37 °C for 30 min, followed by measurement of absorbance at 517 nm using a UV spectrophotometer. 2) PTIO (2‐phenyl‐4,4,5,5‐tetramethylimidazoline‐1‐oxo 3‐oxide, Aladdin, Shanghai, China): A 0.25 μg mL^−1^ solution was prepared using ddH_2_O and mixed with the sample at a 1:1 volume ratio. Absorbance was measured at 557 nm after 2 h of reaction at room temperature. 3) ABTS: A mixed solution containing 7.4 mm ABTS and 2.6 mm K_2_S_2_O_8_ was prepared as the reaction solution and incubated overnight. A volume ratio of 1:3 between the test material solution and reaction solution was used, incubated at room temperature for 10 min, followed by measurement of absorbance at 734 nm.

##### Cytotoxicity and Proliferation Assay

HUVEC and mouse embryonic fibroblasts (NIH3T3) were purchased from ATCC, China. Both cell types were cultured in Dulbecco's Modified Eagle Medium (DMEM) containing 10% fetal bovine serum (FBS) and 1% antibiotics (Gibco, USA). Cell culture was maintained in a 37 °C, 5% CO_2_ incubator (Thermo, USA). Cells in logarithmic growth phase were trypsinized, and 1 × 10^5^ cells were seeded per well in 96‐well plates. Edge wells were filled with phosphate‐buffered saline (PBS) to minimize evaporation. Cells were incubated at 37 °C with 5% CO_2_ for 24 h for attachment. Concentrations for cytotoxicity experiments were set at 0, 1, 10, 50, and 150 μg mL^−1^, while 10 μg mL^−1^ was used for proliferation experiments. After 24 h incubation, the drug‐containing medium was removed and replaced with 100 μL fresh medium. 10 μL MTT solution (final concentration 0.5 μg mL^−1^) was added and gently mixed. Plates were incubated in the dark at 37 °C for 4 h. Liquid was removed, and 100 μL DMSO was added to dissolve crystals. Absorbance (OD) was measured at 490 nm. Cell viability (%) was calculated as: $\left[\frac{\left({\text{OD}}_{\text{experimental}}-{\text{OD}}_{\text{blank}}\right)}{\left({\text{OD}}_{\text{negative}}-{\text{OD}}_{\text{blank}}\right)}\right]\times 100\%$.

##### Cell Viability Staining and Actin Staining

An appropriate concentration of cell suspension was added to 24‐well plates and incubated for 24 h. Materials were added at 10 μg mL^−1^ and cocultured for another 24 h. Staining was performed using a Live/Dead Viability/Cytotoxicity Assay Kit (Beyotime, China). Cells were washed twice with PBS, fixed with 4% paraformaldehyde for 15 min at room temperature, and washed two to four times with PBS containing 0.1% Triton X‐100. Actin‐Tracker (1:200 dilution in PBS with 3% BSA and 0.1% Triton X‐100) was added (200 μL/well) and incubated in the dark for 1 h. Cells were washed three times, stained with DAPI for 15 min, and observed using a fluorescence microscope (Olympus, Japan).

##### Cell Migration Assay

NIH3T3 cells were seeded in 6‐well plates and cultured until 90% confluent. Scratches were created using a 200 μL pipette tip. Cells were rinsed three times with PBS and cultured in serum‐free medium containing 10 μg mL^−1^ material for 24 h. Scratch areas were measured using ImageJ. Migration rate (%) was calculated as: $\frac{\left(\text{Initial}\;\;\text{area}\;-\;\text{Area}\;\text{at}\;\text{time}\;t\right)}{\text{Initial}\;\;\text{area}}\times 100\%$.

##### Tube Formation Assay

A 96‐well plate and accessories were precooled on ice. 60 μL Matrigel was added to each well and incubated at 37 °C for 30 min. 1 × 10^4^ HUVEC cells/well were cultured for 4 h. Tubular structures were analyzed using ImageJ.

##### Cell Polarization Experiment

RAW264.7 cells (5 × 10^5^/well) were seeded in 6‐well plates. After 24 h, M1 polarization was induced with LPS (100 ng mL^−1^) and IFN‐?‐γ (20 ng mL^−1^). Cells were treated with 10 μg mL^−1^ material for 24 h, harvested, and stained with CD86/CD206 antibodies for flow cytometry.

##### Antibacterial Experiment

*S. aureus* (ATCC 25 923) and *E. coli* (ATCC 25 922) were cultured in LB medium. Monoclonal colonies were selected and cultured for 6 h. Bacterial concentration was adjusted to 1 × 10^8^ CFU mL^−1^ in saline containing 150 μg mL^−1^ MS@Cu. After 24 h incubation, 50 μL bacterial suspension was spread on agar plates and incubated at 37 °C. Remaining bacteria were stained using a Live/Dead kit and imaged by confocal microscopy.

##### Intracellular ROS Clearance

NIH3T3 cells were seeded in 6‐well plates at a density of 1 × 10^5^ cells per well and cultured for 24 h in DMEM supplemented with 10% FBS at 37 °C under 5% CO_2_. After the incubation period, the cells were treated with MS@Cu0.10 (at a concentration of 10 μg mL^−1^) and 100 μm H_2_O_2_. Intracellular ROS levels were detected using an ROS Assay Kit (Beyotime, China), following the manufacturer's instructions. Briefly, after treatment, cells were incubated with 10 μm 2′,7′‐dichlorofluorescin diacetate (DCFH‐DA) at 37 °C for 30 min. Subsequently, the cells were washed three times with PBS to remove any residual probe. Fluorescence images were captured using an inverted fluorescence microscope, and the fluorescence intensity was quantified with ImageJ software (National Institutes of Health, Bethesda, MD, USA).

##### Animal Model Construction

Diabetic SD rat models were established by intraperitoneal injection of streptozotocin (70 mg kg^−1^). Rats with blood glucose ≥16.7 mm were selected. Skin defects were created under isoflurane anesthesia. A 10‐mm circular full‐thickness defect was generated. 10 CFU mL^−1^ *S. aureus* was applied to wounds. Rats were divided into four groups: blank, MN patch, Mg_2_SiO_4_‐loaded patch, and MS@Cu0.1‐loaded patch. Wounds were photographed every 2 days. On day 11, rats were euthanized by cervical dislocation after anesthesia. Skin tissues were fixed, embedded, and stained with H&E, Masson, and antibodies (IL‐1β/IL‐10). All procedures were approved by the Shanghai Tenth People's Hospital Ethics Committee (SHDSYY‐2024‐4494).

##### RNA Sequencing Analysis

Two sample sets were prepared: NIH3T3 cells and MS@Cu‐treated cells. RNA was extracted and analyzed using the Illumina TruseqTM RNA kit (Majorbio Biotech). Sequencing data were analyzed via the Majorbio cloud platform.

##### Western Blot

NIH3T3 cells were seeded in 6‐well plates and cultured to 80%–90% confluence. Cells were treated with MS@Cu0.01/0.05/0.1 for 24 h. Proteins were extracted using RIPA buffer with protease/phosphatase inhibitors. Electrophoresis and transfer were performed (70 V, 70 min). Membranes were blocked for 30 min, incubated with primary antibodies (PI3K/p‐PI3K, AKT/p‐AKT) overnight, and detected using fluorescent secondary antibodies with Odyssey Scanner.

##### Statistical Analysis

The data were presented as mean±standard deviation (SD). Each experiment was repeated at least three times and measured in parallel (n ≥ 3). The statistical differences were determined by unpaired Student's t‐test and one‐way analysis of variance using the statistical product and service solutions software (version 10.1.2, Prism). A difference of *p* < 0.05 was considered to be statistically significant, and that of *p* < 0.01 or *p* < 0.001 was considered to be highly significant.

## Supporting Information

Supporting Information is available from the Wiley Online Library or from the author.

## Conflict of Interest

The authors declare no conflict of interest.

## Supporting information

Supplementary Material

## Data Availability

The data that support the findings of this study are available on request from the corresponding author. The data are not publicly available due to privacy or ethical restrictions.
