# Supplementary material for: Copper Nanocluster‐Decorated Magnesium Silicate‐Based Microneedle Enhances Antimicrobial Effects and Tissue Remodeling for Diabetic Wounds
Source: Small Sci. 2025 Nov 19;6(1):e202500442. doi: 10.1002/smsc.202500442 (PMC12798781; doi:10.1002/smsc.202500442)
Supplement: Supplementary file 1 — Supplementary Material [file SMSC-6-e202500442-s001.pdf]

Supporting information

Copper Nanocluster-Decorated Magnesium Silicate based Microneedle Enhances Antimicrobial effects and Tissue Remodeling for Diabetic Wounds

Shuo Tan[1,†], Hua Zeng[1,†], Wenshuya Li[2,†], Haibo Liu[1], Xuefeng Gu[3,*], Xiong Luo[4,*], Xinyu Zhao[1,*]

[1]Center for Orthopaedic Science and Translational Medicine, Department of Orthopaedics, Shanghai Tenth People's Hospital, School of Medicine, Tongji University, Shanghai 200072, P. R. China

[2]Department of Plastic Surgery, The Second Hospital of Hebei Medical University, Shijiazhuang, Hebei, P. R. China

[3]Shanghai Key Laboratory of Molecular Imaging, School of Pharmacy, Shanghai University of Medicine & Health Sciences, Shanghai 201318, P. R. China

[4]Department of Joint and Sports Medcine, Shanghai Fourth People's Hospital, School of Medicine, Tongji University, Shanghai 200434, P. R. China

*Corresponding author: E-mail: guxf@sumhs.edu.cn, 2305179@tongji.edu.cn, xyzhao@tongji.edu.cn

†Authors contributed equally.

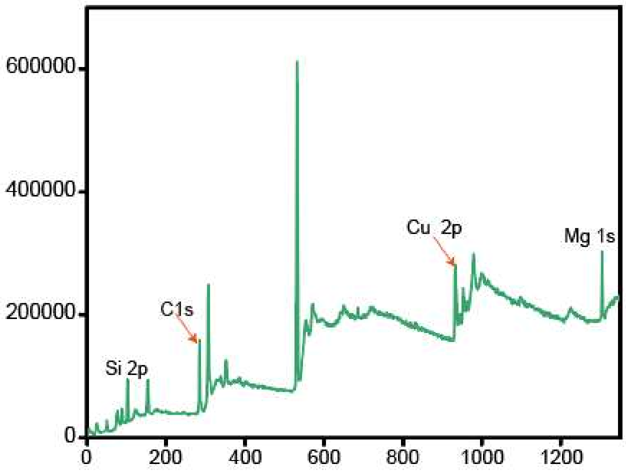

Figure S1. The XPS spectrum of MS@Cu0.10.

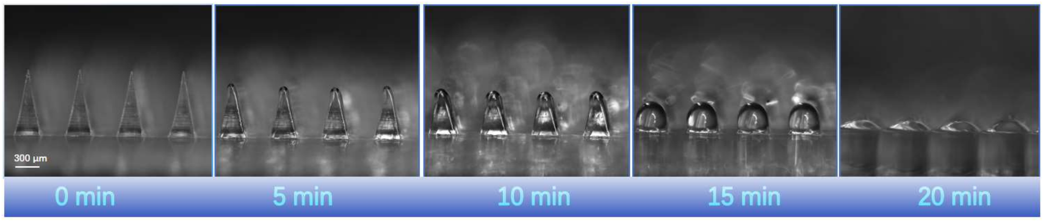

Figure S2. Ablation images of microneedles at different time points in a 75% humidity environment.

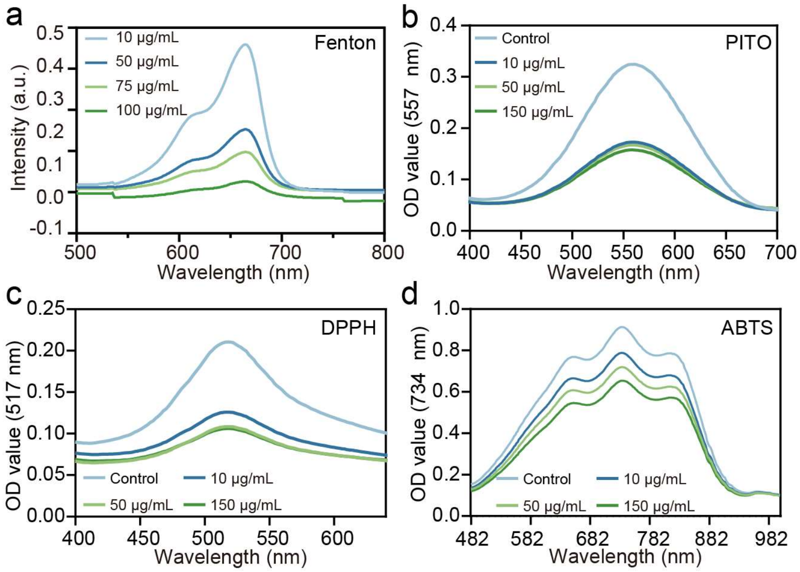

Figure S3. Fenton reaction activity and reactive oxygen species (ROS) scavenging

capacity of MS@Cu0.10. (a) Fenton reaction kinetics mediated by MS@Cu0.10 at concentrations ranging from 0 to 150 μg/mL (incubation time: 24 h; substrate: H_2O_2). (b–d) ROS scavenging effects detected in cells treated with MS@Cu0.10 at 0, 10, 50, and 150 μg/mL, respectively.

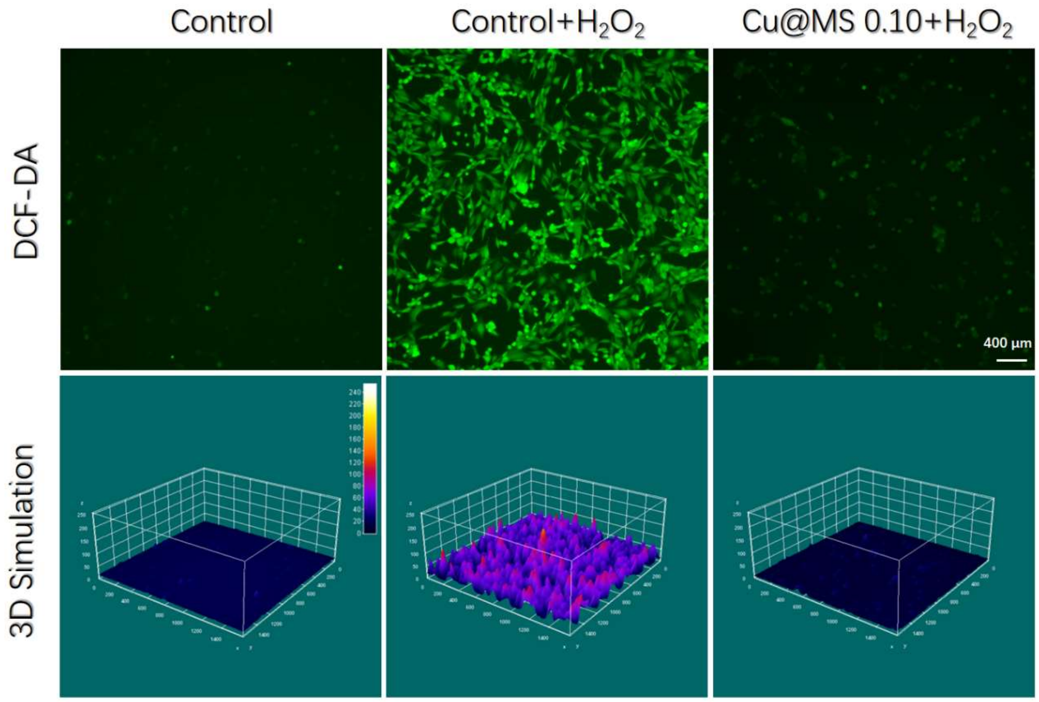

Figure S4. Representative images of intracellular ROS staining and corresponding three-dimensional (3D) fluorescence reconstruction in NIH3T3 cells under different treatment conditions: untreated control, H_2O_2 treatment, and MS@Cu0.10 + H_2O_2 co-treatment.
